# Epidemiologic Trends of Dengue in U.S. Territories, 2010–2020

**DOI:** 10.15585/mmwr.ss7204a1

**Published:** 2023-05-19

**Authors:** Kyle R. Ryff, Aidsa Rivera, Dania M. Rodriguez, Gilberto A. Santiago, Freddy A. Medina, Esther M. Ellis, Jomil Torres, Ann Pobutsky, Jorge Munoz-Jordan, Gabriela Paz-Bailey, Laura E. Adams

**Affiliations:** ^1^Division of Vector-borne Diseases, CDC, San Juan, Puerto Rico; ^2^Virgin Islands Department of Health, ^3^Puerto Rico Department of Health, ^4^Guam Department of Public Health and Social Services

## Abstract

**Problem/Condition:**

Dengue is one of the most common vectorborne flaviviral infections globally, with frequent outbreaks in tropical regions. In 2019 and 2020, the Pan American Health Organization reported approximately 5.5 million dengue cases from the Americas, the highest number on record. In the United States, local dengue virus (DENV) transmission has been reported from all U.S. territories, which are characterized by tropical climates that are highly suitable for *Aedes* species of mosquitoes, the vector that transmits dengue. Dengue is endemic in the U.S. territories of American Samoa, Puerto Rico, and the U.S. Virgin Islands (USVI). Dengue risk in Guam and the Commonwealth of the Northern Mariana Islands is considered sporadic or uncertain. Despite all U.S. territories reporting local dengue transmission, epidemiologic trends over time have not been well described.

**Reporting Period:**

2010–2020.

**Description of System:**

State and territorial health departments report dengue cases to CDC through ArboNET, the national arboviral surveillance system, which was developed in 2000 to monitor West Nile virus infections. Dengue became nationally notifiable in ArboNET in 2010. Dengue cases reported to ArboNET are categorized using the 2015 Council of State and Territorial Epidemiologists case definition. In addition, DENV serotyping is performed at CDC’s Dengue Branch Laboratory in a subset of specimens to support identification of circulating DENV serotypes.

**Results:**

During 2010–2020, a total of 30,903 dengue cases were reported from four U.S. territories to ArboNET. Puerto Rico reported the highest number of dengue cases (29,862 [96.6%]), followed by American Samoa (660 [2.1%]), USVI (353 [1.1%]), and Guam (28 [0.1%]). However, annual incidence rates were highest in American Samoa with 10.2 cases per 1,000 population in 2017, followed by Puerto Rico with 2.9 in 2010 and USVI with 1.6 in 2013. Approximately one half (50.6%) of cases occurred among persons aged <20 years. The proportion of persons with dengue who were hospitalized was high in three of the four territories: 45.5% in American Samoa, 32.6% in Puerto Rico, and 32.1% in Guam. In Puerto Rico and USVI, approximately 2% of reported cases were categorized as severe dengue. Of all dengue-associated deaths, 68 (0.2%) were reported from Puerto Rico; no deaths were reported from the other territories. During 2010–2020, DENV-1 and DENV-4 were the predominant serotypes in Puerto Rico and USVI.

**Interpretation:**

U.S. territories experienced a high prevalence of dengue during 2010–2020, with approximately 30,000 cases reported, and a high incidence during outbreak years. Children and adolescents aged <20 years were disproportionately affected, highlighting the need for interventions tailored for this population. Ongoing education about dengue clinical management for health care providers in U.S. territories is important because of the high hospitalization rates reported. Dengue case surveillance and serotyping can be used to guide future control and prevention measures in these areas.

**Public Health Action:**

The Advisory Committee on Immunization Practices recommends vaccination with Dengvaxia for children aged 9–16 years with evidence of previous dengue infection and living in areas where dengue is endemic. The recommendation for the dengue vaccine offers public health professionals and health care providers a new intervention for preventing illness and hospitalization in the age group with the highest burden of disease in the four territories (Paz Bailey G, Adams L, Wong JM, et al. Dengue Vaccine: Recommendations of the Advisory Committee on Immunization Practices, United States, 2021. MMWR Recomm Rep 2021;70[No. RR-6]). American Samoa, Puerto Rico, and USVI are all considered endemic areas and persons residing in these areas are eligible for the new dengue vaccine. Persons aged 9–16 years in those jurisdictions with laboratory evidence of previous dengue infection can receive the dengue vaccine and benefit from a reduced risk for symptomatic disease, hospitalization, or severe dengue. Health care providers in these areas should be familiar with the eligibility criteria and recommendations for vaccination to reduce the burden of dengue among the group at highest risk for symptomatic illness. Educating health care providers about identification and management of dengue cases can improve patient outcomes and improve surveillance and reporting of dengue cases.

## Introduction

Dengue is a flavivirus transmitted by infected *Aedes* mosquitos, including *Aedes aegypti* and *Aedes albopictus*. Dengue remains one of the most common mosquito-transmitted viral infections globally, with growing numbers of cases in recent years. In 2019 and 2020, approximately 5.5 million dengue cases were reported to the Pan American Health Organization from the Americas, the highest number on record (*1*). In the United States, local dengue virus (DENV) transmission has been reported from all U.S. territories (*2*), which are characterized by tropical climates that are highly suitable for *Aedes* species mosquitoes that transmit dengue. Dengue is endemic, defined as having >10 cases in at least three of the previous 10 years, in the U.S. territories of American Samoa, Puerto Rico, and the U.S. Virgin Islands (USVI). Dengue risk in the Commonwealth of the Northern Mariana Islands (CNMI) and Guam is considered sporadic or uncertain. Persons living in or traveling to areas where dengue is endemic are at risk for dengue infection and potentially severe, life-threatening disease. Severe dengue is defined as dengue with any of the following: 1) severe plasma leakage as demonstrated by hypovolemic shock, extravascular fluid accumulation with respiratory distress, and/or hemoconcentration; 2) severe bleeding from the gastrointestinal tract or vagina requiring medical intervention, including intravenous fluid resuscitation or blood transfusion; or 3) severe organ involvement including any of the following elevated liver transaminases: aspartate aminotransferase or alanine aminotransferase ≥1,000/L; impaired level of consciousness and/or diagnosis of encephalitis, encephalopathy, or meningitis; and/or heart or other organ involvement including myocarditis, cholecystitis, and pancreatitis (*3*).

The increased prevalence and geographic expansion of dengue in recent years has been a challenge because of the limited tools available for prevention and control and lack of specific antivirals for treatment. However, in June 2021, the Advisory Committee on Immunization Practices (ACIP) recommended use of Dengvaxia dengue vaccine to prevent illness, hospitalization, and severe disease from dengue among children aged 9–16 years with laboratory-confirmed previous DENV infection and living in areas of the United States where dengue is endemic (*4*,*5*). However, understanding the burden of dengue among eligible populations is a critical consideration when implementing a new vaccine program and evaluating its potential effectiveness. Although several of the U.S. territories are listed as eligible for the vaccine, little information is available about recent dengue trends in those areas, circulating serotypes, or specific groups affected. This report describes the characteristics of dengue cases and trends in U.S. territories during 2010–2020. This information is intended to help public health professionals and health care providers adapt dengue prevention strategies in U.S. territories for groups of persons that experience a high burden of disease caused by dengue.

## Methods

State and territorial health departments report dengue cases to CDC through ArboNET, the national arboviral surveillance system, which was developed in 2000 to monitor West Nile virus infections in humans, mosquitoes, birds, and other animals. Dengue surveillance in Puerto Rico has a long history beginning with the first report of dengue in 1899 and continuing with sporadic reports of dengue’s presence in the island until 1963, when the Puerto Rico Department of Health (PRDH) requested assistance from CDC to investigate a large outbreak. PRDH again requested assistance in 1969 during a DENV-2 outbreak. During this same year, the Passive Dengue Surveillance System was initiated to collect demographic and clinical data on dengue cases for the characterization of dengue in Puerto Rico (*6*). This system is still used to collect data on Puerto Rico’s dengue cases, which are reported to ArboNET.

Puerto Rico and USVI are unincorporated territories of the United States located in the Caribbean Sea. Puerto Rico includes one main island and two smaller islands and has the largest population of the U.S. territories with approximately 3.3 million residents according to the 2020 U.S. census (*7*). USVI comprises three islands and has a population of approximately 87,000 persons (*8*). American Samoa is an unincorporated territory of the United States in the South Pacific Ocean and comprises five islands with a population of approximately 50,000 persons (*9*). Guam is an unincorporated territory of the United States in the Western Pacific Ocean with approximately 154,000 persons (*10*). CNMI did not report locally acquired dengue cases to ArboNET during the surveillance period and is excluded from this analysis.

Dengue became nationally notifiable in ArboNET in 2010 (*11*). This designation, which is established by the Council of State and Territorial Epidemiologists (CSTE) and CDC for diseases and conditions of public health importance, allows states and territories to voluntarily inform CDC of persons meeting case criteria for diseases and conditions that have been identified with the nationally notifiable designation (https://www.cdc.gov/nndss/about/index.html). Dengue cases reported to ArboNET were categorized using the 2015 CSTE case definition (*3*). Confirmed cases met the clinical criteria and had detection of 1) DENV nucleic acid by RT-PCR in any body fluid or tissue, 2) DENV antigen in tissue by a validated assay, 3) DENV NS1 antigen by a validated immunoassay, or 4) immunoglobulin M (IgM) anti-DENV antibody if exposure occurred in an area without evidence of other flavivirus transmission. Probable dengue cases met the clinical criteria and were defined by detection of IgM anti-DENV antibody in serum if the person lived in or traveled to an area with transmission of another flavivirus.

Dengue clinical criteria is defined as fever as reported by the patient or health care provider and the presence of one or more of the following signs and symptoms: nausea or vomiting; rash; aches and pains; tourniquet test positive (https://www.cdc.gov/dengue/training/cme/ccm/Tourniquet%20Test_F.pdf), which is a marker of capillary fragility; leukopenia (a total white blood cell count of <5,000/mm^3^); any warning sign for severe dengue such as abdominal pain or tenderness, persisting vomiting, pleural or pericardial effusion, ascites, or mucosal bleeding at any site; liver enlargement >2 cm; or increasing hematocrit concurrent with rapid decrease in platelet count. Dengue-like illness is defined as fever without other symptoms.

Cases were described by selected characteristics and estimated dengue incidence per 1,000 population using 2020 U.S. census population data (*7*–*10*). To provide a more complete picture of circulating DENVs, additional serotype data from dengue cases reported directly to or tested by CDC Dengue Branch laboratory were assessed from American Samoa, Puerto Rico, and USVI. The infecting DENV was determined by molecular typing by RT-PCR (*12*–*14*).

## Results

During 2010–2020, a total of 30,903 cases were reported to ArboNET from American Samoa, Guam, Puerto Rico, and USVI; 21,705 (70.2%) were confirmed and 9,198 (29.8%) were probable (Table 1). The highest number of dengue cases occurred among persons aged <20 years, accounting for approximately half (15,640 [50.6%]) of reported cases. A majority of cases occurred in males, accounting for 16,808 (54.4%) of all cases. Approximately 2% (584) of all cases were categorized as severe dengue. A total of 10,037 (32.4%) persons with dengue were hospitalized, and 68 (0.2%) deaths were reported. Travel to a country with local transmission of dengue in the 2 weeks before symptom onset was reported in 28 (0.1%) cases.

**TABLE 1:** Number and percentage of dengue cases, by selected characteristics and U.S. territory — ArboNET, 2010–2020

Characteristic	Puerto Rico No. (%)	American Samoa No. (%)	U.S. Virgin Islands No. (%)	Guam No. (%)	Total No. (%)
**Case status**					
Confirmed	20,675 (69.2)	660 (100)	342 (96.9)	28 (100)	**21,705 (70.2)**
Probable	9,187 (30.8)	0 (—)	11 (3.1)	0 (—)	**9,198 (29.8)**
**Sex**					
Female	13,557 (45.4)	332 (50.3)	188 (53.3)	11 (39.3)	**14,088 (45.6)**
Male	16,305 (54.6)	328 (49.7)	158 (44.8)	17 (60.7)	**16,808 (54.4)**
Unknown	0 (—)	0 (—)	7 (2.0)	0 (—)	**7 (**—**)**
**Age group (yrs)**					
0–9	3,924 (13.1)	146 (22.1)	36 (10.2)	2 (7.1)	**4,108 (13.3)**
10–19	11,146 (37.3	303 (45.9)	76 (21.5)	7 (25.0)	**11,532 (37.3)**
20–29	4,471 (15.0)	78 (11.8)	53 (15.0)	3 (10.7)	**4,605 (14.9)**
30–39	2,574 (8.6)	43 (6.5)	38 (10.8)	6 (21.4)	**2,661 (8.6)**
40–49	2,206 (7.4)	40 (6.1)	41 (11.6)	3 (10.7)	**2,290 (7.4)**
50–59	2,146 (7.2)	23 (3.5)	41 (11.6)	4 (14.3)	**2,214 (7.2)**
60–69	1,797 (6.0)	16 (2.4)	42 (11.9)	3 (10.7)	**1,858 (6.0)**
≥70	1,195 (4.0)	11 (1.7)	18 (5.1)	0 (—)	**1,224 (4.0)**
Unknown	403 (1.3)	0 (—)	8 (2.3)	0 (—)	**411 (1.3)**
**Clinical syndrome**					
Dengue	23,217 (77.7)	660 (100)	325 (92.1)	28 (100)	**24,230 (78.4)**
Dengue-like illness	90 (0.3)	0 (—)	18 (5.1)	0 (—)	**108 (0.3)**
Other clinical	43 (0.1)	0 (—)	1 (0.3)	0 (—)	**44 (0.1)**
Severe dengue	578 (1.9)	0 (—)	6 (1.7)	0 (—)	**584 (1.9)**
Unknown	5,934 (19.9)	0 (—)	3 (0.8)	0 (—)	**5,937 (19.2)**
**Outcome**					
Hospitalized	9,724 (32.6)	300 (45.5)	3 (0.8)	9 (32.1)	**10,037 (32.4)**
Died	68 (0.2)	0 (—)	0 (—)	0 (—)	**68 (0.2)**
**Import status**					
Travel associated	14 (—)	1 (0.2)	0 (—)	13 (46.4)	**28 (0.1)**
Locally acquired	29,848 (100)	659 (99.8)	352 (99.7)	15 (53.6)	**30,874 (99.9)**
Unknown	0 (—)	0 (—)	1 (0.3)	0 (—)	**1 (**—**)**
**Total**	**29,862 (96.6)**	**660 (2.1)**	**353 (1.1)**	**28 (0.1)**	**30,903 100**

### Puerto Rico

**FIGURE 1:**
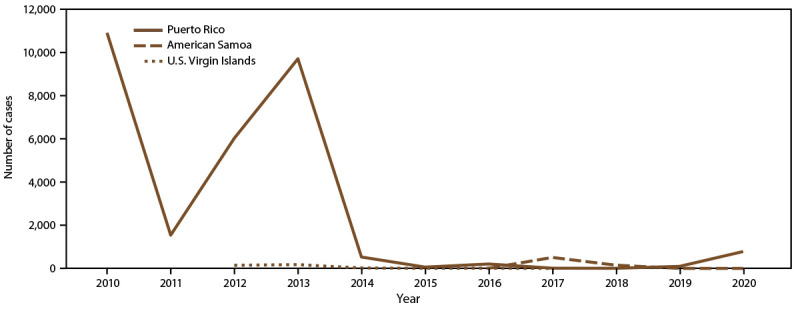
Number of dengue cases, by U.S. territory and year of onset, 2010–2020* * Puerto Rico: N = 29,862; American Somoa: N = 660; U.S. Virgin Islands: N = 353.

**Source:** ArboNet.

**FIGURE 2:**
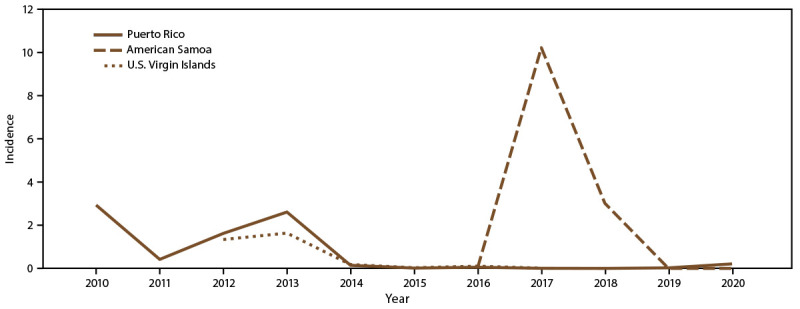
Annual incidence* of dengue cases, by U.S. territory and year of onset, 2010–2020^†^ * Per 1,000 population using 2020 U.S. census population data. ^†^ Puerto Rico: N = 29,862; American Somoa: N = 660; U.S. Virgin Islands: N = 353.

**Source:** ArboNet.

**FIGURE 3:**
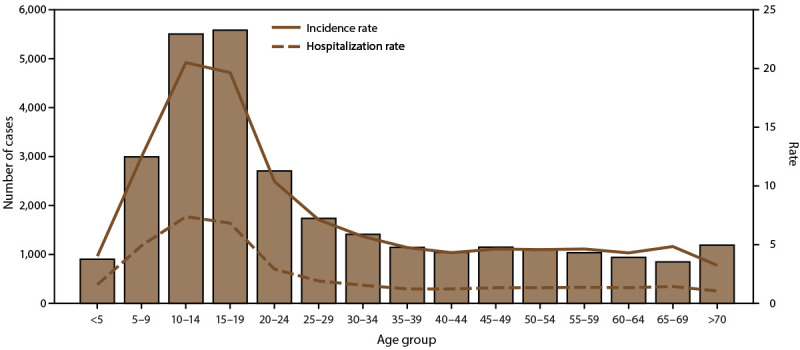
Number of dengue cases, incidence rate,* and hospitalization rate, by age group — Puerto Rico, 2010–2020^†^ * Per 1,000 population using 2020 U.S. census population data. ^†^ N = 29,862.

**Source:** ArboNet.

**FIGURE 4:**
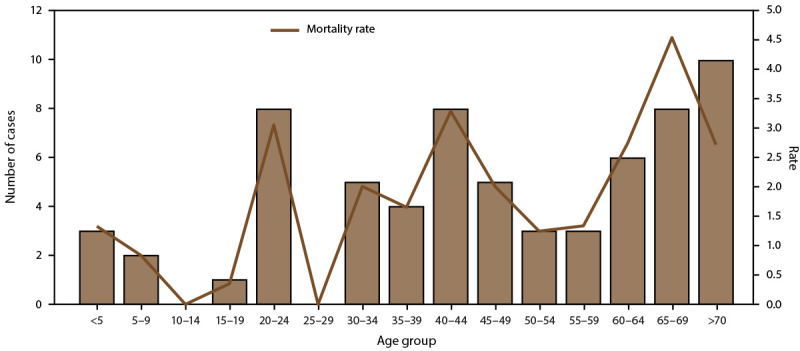
Number of fatal dengue cases and mortality rate,* by age group — Puerto Rico, 2010–2020^†^ * Per 1,000 population using 2020 U.S. census population data. ^†^ N = 29,862.

**Source:** ArboNet.

During 2010–2020, the majority (29,862 [96.6%]) of dengue cases were reported from Puerto Rico (Figure 1). Annual incidence per 1,000 population was highest during the two outbreaks in 2010 and 2013, 2.9 and 2.6, respectively (Figure 2). Among all reported cases from Puerto Rico, 20,675 (69.2%) were confirmed and 9,187 (30.8%) were probable; 54.6% of dengue cases were in males. Approximately 50% of reported cases in Puerto Rico occurred among persons aged <20 years, with the 10–19-year-old age group accounting for 37.3% of cases. The highest incidence and hospitalization rates also occurred among children aged 10–14 and 15–19 years (Figure 3). In Puerto Rico, 32.6% of persons with dengue were hospitalized and 68 dengue-associated deaths were reported during 2010–2020; the highest number of deaths (n = 10) occurred among persons aged >70 years, although six (8.8%) of the deaths occurred among persons aged <20 years (Figure 4). Almost all (>99%) cases of dengue in Puerto Rico were locally acquired.

### American Samoa

**FIGURE 5:**
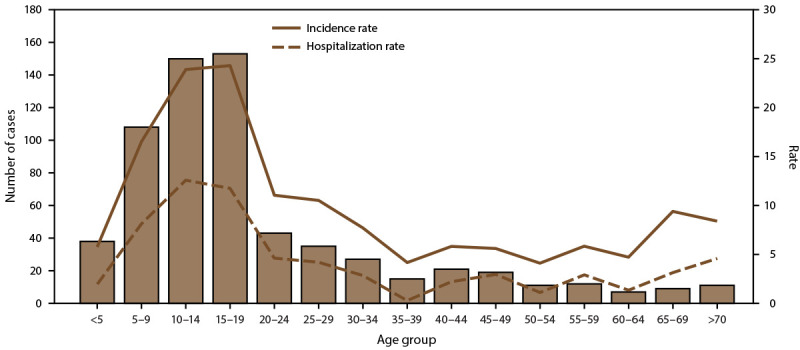
Number of dengue cases, incidence rate,* and hospitalization rate, by age group — American Samoa, 2010–2020^†^ * Per 1,000 population using 2020 U.S. census population data. ^†^ N = 660.

**Source:** ArboNet.

During 2010–2020, American Samoa accounted for 660 (2.1%) of all reported cases in the U.S. territories (Figure 1). Annual incidence per 1,000 population was highest in 2017, reaching 10.2 per 1,000 population (Figure 2). All dengue cases were confirmed, and 50.3% were in females. The highest case numbers and rates occurred among persons aged <20 years; 68.0% of reported cases occurred among persons aged <20 years, and 45.9% occurred among persons aged 10–19 years. The proportion of persons with dengue cases hospitalized in American Samoa (45.5%) was similar to Puerto Rico (Table 1); the highest incidence rates and hospitalizations occurred in the 10–14 and 15–19-year-old age groups (Figure 5). No dengue-associated deaths were reported, and one travel-associated case was reported.

### U.S. Virgin Islands

**FIGURE 6:**
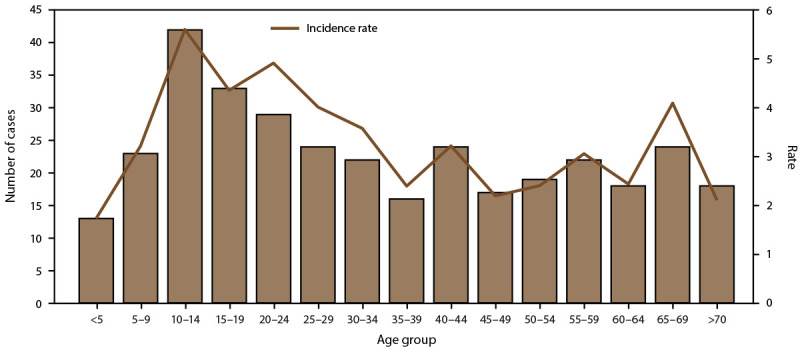
Number of dengue cases and incidence rate* — U.S. Virgin Islands, 2010–2020^†^ * Per 1,000 population using 2020 U.S. census population data. ^†^ N = 353.

**Source:** ArboNet.

During 2010–2020, USVI accounted for 353 (1.1%) of all reported cases in the U.S. territories (Figure 1). Annual incidence per 1,000 population was highest during the 2012–2013 outbreak with an annual incidence of 1.6 in 2013 (Figure 2). Almost all (96.9%) reported cases in USVI were confirmed, and 53.3% were in females. Approximately one third of reported cases occurred among children and adolescents aged <20 years, with a majority (21.5%) in the 10–19-year-old age group. The 20–29-year-old age group was the second most affected age group with 15.0% of cases. Three (0.8%) persons with dengue were hospitalized, and no dengue-associated deaths were reported. The highest incidence rate occurred among children aged 10–14 years (Figure 6). All reported cases were locally acquired.

### Guam

During 2010–2020, Guam accounted for 28 (0.1%) of all reported cases in the U.S. territories. All cases were confirmed and occurred in 2019 and 2020; however, nearly one half of all reported cases (13 [46.4%]) were travel associated. More than one half (60.7%) of cases were in males. Similar to the other U.S. territories, the 10–19-year-old age group was most affected, with approximately 25% of reported cases. Unlike the other territories, the next most affected age group was persons aged 30–39 years, accounting for 21.4% of cases. Thirty-two percent of persons with dengue were hospitalized, and no dengue-associated deaths were reported (Table 1).

### Serotype Distribution in Puerto Rico, USVI, and American Samoa

**FIGURE 7:**
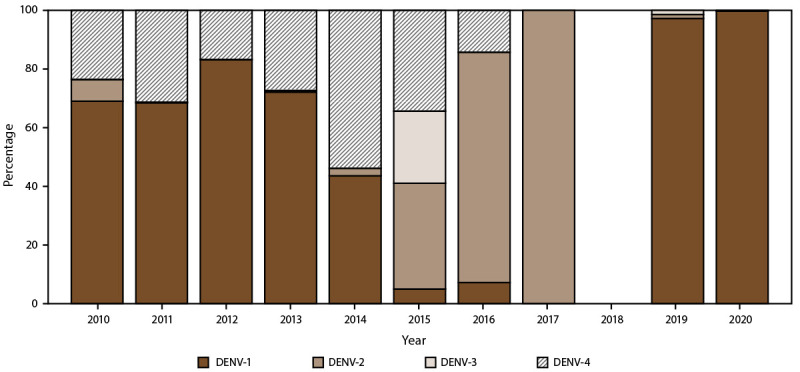
Percentage of dengue cases, by serotypes and year — Puerto Rico, 2010–2020* **Abbreviation:** DENV = dengue virus. * N = 29,862.

**Source:** ArboNet.

A total of 21,329 confirmed cases from Puerto Rico (21,194), USVI (119), and American Samoa (16) had DENV serotype data reported to or performed by the CDC Dengue Branch Laboratory (Table 2). In Puerto Rico, the majority (72.9%) of cases were DENV-1, followed by DENV-4 (24.1%), which were associated with the large dengue outbreaks during 2010–2013 (Figure 7). Concurrent with the emergence of chikungunya in 2014 and Zika in 2016, overall dengue case counts decreased from 2015 to 2019, with all four DENV serotypes circulating at low levels. In 2019 and 2020, dengue activity increased slightly, with all locally acquired cases identified as DENV-1, in addition to multiple travel-associated cases of DENV-2 and DENV-3. In USVI, DENV-1 and DENV-4 were the two circulating dengue serotypes during the outbreaks during 2012–2013, with DENV-1 accounting for 80.7% of cases with serotype available. In American Samoa, limited serotype data were available, although DENV-2 (n = 2) and DENV-3 (n = 14) cases were reported.

**TABLE 2:** Number and percentage of dengue virus (DENV) serotype, by U.S. territory — ArboNET, 2010–2020

Serotype	Puerto Rico No. (%)	U.S. Virgin Islands No. (%)	American Samoa No. (%)
DENV-1	15,442 (72.9)	96 (80.7)	0 (—)
DENV-2	637 (3.0)	2 (1.7)	2 (12.5)
DENV-3	5 (—)	1 (0.8)	14 (87.5)
DENV-4	5,110 (24.1)	20 (16.8)	0 (—)
**Total**	**21,194 (100)**	**119 (100)**	**16 (100)**

## Discussion

U.S. territories experienced a high prevalence of dengue during 2010–2020, with approximately 30,000 confirmed and probable cases reported. Approximately one half of the cases occurred among persons aged <20 years, with the highest rate of cases and hospitalizations among children aged 10–19 years. A majority of (96.6%) cases were reported from Puerto Rico, which also was the only territory to report dengue-associated deaths. Although cases from American Samoa were only reported during 2017–2018, this jurisdiction also had the highest annual incidence of dengue cases among the territories, with 10.2 cases per 1,000 population in 2017. Similarly, in USVI, the majority of cases were reported during a short period (2012–2013), during which the jurisdiction experienced high rates of dengue. The proportion of persons hospitalized varied by jurisdiction, with nearly one half of those in American Samoa hospitalized, approximately one in three in Puerto Rico and Guam hospitalized, and <1% in USVI hospitalized.

These findings highlight the burden of disease, particularly among children, in U.S. territories with endemic dengue transmission. However, the recommendation for the dengue vaccine offers a new option for preventing illness and hospitalization in the population at greatest risk. American Samoa, Puerto Rico, and USVI are all considered endemic areas eligible for the new dengue vaccine, and persons aged 9–16 years in those jurisdictions with evidence of previous dengue infection can receive the dengue vaccine and benefit from a reduced risk for symptomatic disease, hospitalization, or severe dengue (*4*,*5*,*15*). Health care providers in these areas should be familiar with recommendations for testing patients for previous dengue infection before vaccination (https://www.cdc.gov/vaccines/vpd/dengue/hcp/recommendations.html).

Dengue case counts in all the territories were intermittent, with high case numbers reported in outbreak years and few or no cases reported in other years. This was particularly true for American Samoa, USVI, and Guam, which had no cases reported in 8, 4, and 9 years of the 10-year period, respectively. Puerto Rico had low levels of endemic dengue transmission after the outbreaks during 2010–2013, with case numbers at historically low levels during 2016–2019 compared with previous years. This could be associated with the introduction of Zika virus in late 2015 and short-term cross-protection against DENV, as suggested by findings from other countries in Latin America (*16*). None of the jurisdictions with endemic dengue transmission experienced higher than average numbers in 2019, in contrast to other regions such as Central and South America, which had the highest number of dengue cases reported on record (*1*). In 2019, Guam experienced its first locally acquired dengue cases since 1944, concurrent with widespread dengue transmission in the Pacific and Western Pacific regions (*17*,*18*), although transmission was not widespread across the island. The difference in outbreak risk might be attributable to the mosquito vector. *Ae. albopictus* is the primary dengue vector in Guam and *Ae. aegypti* in the other territories.

Identifying dengue cases and the circulating DENV serotypes in affected regions can improve understanding of transmission patterns and susceptibility within populations to different DENV serotypes. DENV-1 was predominant in outbreaks in the Caribbean during 2010–2020, and low levels of circulation by the other three DENVs could indicate increased population-level susceptibility to DENV serotypes that haven’t circulated in recent years. In comparison, DENV-2 and DENV-3 were the primary viruses identified from American Samoa and Guam during the same period. As population-level immunity to the four DENV serotypes decreases because of relatively low levels of circulation, the need for large-scale vaccine programs, education for health care providers, and integrated vector control strategies based on near real-time epidemiology and laboratory data become increasingly important to decrease the likelihood of large outbreaks and severe outcomes because of dengue.

## Limitations

The findings in this report are subject to at least two limitations. First, case numbers are underestimated because not all symptomatic persons seek health care, receive appropriate testing, or are reported to ArboNET. Publications from American Samoa reported 16 cases of DENV-3 in 2015 (*19*) and 941 cases of DENV-2 during an outbreak between November 2016 and October 2018 (*20*); however, only partial cases were reported to ArboNET and included in the results. This probably resulted in lower incidence rates and hospitalization numbers for American Samoa. Second, a relatively small number of severe dengue cases were identified from all areas, which could be because of incomplete case information for categorization; for example, a large subset of reported cases with unknown clinical outcomes in Puerto Rico might obscure the severity of dengue in reported cases. Data contained in this report likely represent an underreporting of both case numbers and severity.

## Future Directions

Although a large number of cases were identified during the surveillance period, it is likely that these are a substantial underestimate of the true number of dengue cases occurring in the areas where dengue is endemic. Jurisdictions should ensure all dengue cases are reported to ArboNET to harmonize dengue case numbers using standardized definitions across the jurisdictions. Dengue surveillance in U.S. territories could also be strengthened by increasing laboratory testing among suspected cases and performing appropriate testing to identify circulating DENV serotypes, which were reported for <2% of cases in ArboNET during the surveillance period. DENV serotyping can be implemented by state or territorial public health laboratories. Strengthening dengue surveillance in areas where dengue is endemic using standardized case definitions also aligns with regional and national goals to improve dengue case reporting across the Americas, an initiative led by the Pan American Health Organization and supported by CDC to better describe dengue burden and transmission patterns and inform prevention efforts in the region.

Classification of dengue cases by the 2015 DENV infections case definition can be challenging because of the detailed clinical criteria, which require both clinical expertise and access to detailed clinical records to fully verify severe and nonsevere case status. Education of health care providers and arboviral surveillance coordinators could help standardize case classification in areas where dengue is endemic and support clinical management for severe cases. No standardized dengue case surveillance form is in use in the U.S. territories. Implementation of a standardized dengue case surveillance form at the territorial jurisdictional level could improve case surveillance and classification and automation of case reporting to ArboNET. Automated reporting of dengue cases can improve surveillance and help with standardization of dengue case classification.

The findings in this report highlight the high prevalence of disease, particularly among children, in U.S. territories with endemic dengue transmission. Control of *Ae. aegypti* is complicated by cryptic and inaccessible breeding sites that make it difficult to locate and control a large proportion of the targeted mosquito population (*21*). Furthermore, insecticide resistance to *Ae. aegypti* is widespread, and new regulatory requirements have resulted in discontinuation of some insecticides and greater difficulty in registering new chemicals (*22*). Successful broad-scale application of integrated vector control management strategies has been difficult to achieve and sustain. The recommendation for a safe and effective dengue vaccine offers a new option for preventing illness from dengue in the population at greatest risk. The ACIP recommendation of Dengvaxia marks the first time a dengue vaccine has been available in the United States for broad-scale dengue prevention. Takeda and the National Institutes of Health have two vaccines (TAK-003 and TV003, respectively) that are in late-stage vaccine trials (*23*).

## Conclusion

During 2010–2020, the prevalence of dengue was high in American Samoa, Puerto Rico, and USVI, particularly during outbreak years. A localized dengue outbreak occurred in Guam, although this did not meet the criteria for endemic dengue transmission. In all territories, the highest prevalence of disease occurred among persons aged <20 years, highlighting the need for prevention and control activities in this population. American Samoa, Puerto Rico, and USVI are all considered endemic areas eligible for the new dengue vaccine in persons aged 9–16 years with previous dengue infection. Implementation of robust vaccination programs in these areas could prevent illness, hospitalization, and severe disease from dengue in the most severely affected age group. New dengue vaccines will likely expand the population groups eligible for vaccination and the impact of this intervention.
